# A Qualitative Exploration of Barriers to Condom Use among Female Sex Workers in China

**DOI:** 10.1371/journal.pone.0046786

**Published:** 2012-10-08

**Authors:** Wu Jie, Zhou Xiaolan, Lu Ciyong, Eileen Moyer, Wang Hui, Hong Lingyao, Deng Xueqing

**Affiliations:** 1 Department of Medical Statistics and Epidemiology, School of Public Health, Sun Yat-sen University, Guangzhou, China; 2 Amsterdam Institute of Social Science Research, Department of Sociology and Cultural Anthropology, University of Amsterdam, Amsterdam, The Netherlands; UCL Institute of Child Health, University College London, United Kingdom

## Abstract

**Background:**

Sex workers in China continue to engage in unprotected sex acts that put them at risk for contracting HIV (Human Immunodeficiency Virus) and other STIs (Sexually Transmitted Infections). The purpose of this study was to explore women’s work history, the context of sex work, condom use, HIV testing services, and potential barriers to condom use in a sample of FSWs (female sex workers) in Guangzhou, China.

**Methodology/Principal Findings:**

In-depth, semi-structured, face-to-face interviews were conducted with 24 FSWs in Guangzhou, China. Informants were recruited using a purposive sampling technique. Qualitative data were coded and analyzed using NVivo 8.0. The majority of respondents were internal economic migrants who had entered the sex industry in pursuit of greater financial reward. Most women in the study were married or had steady boyfriends, and were young, with secondary education and limited knowledge about HIV and STIs. Most were not satisfied with their current living conditions and expressed a desire to leave the sex industry. Women reported that they were more likely to use condoms during sex acts with commercial partners than with non-commercial partners. The potential stigma of being seen as a sex worker prevented many from accessing HIV testing. Three key factors put these FSWs at risk for HIV and STIs: unreasonable trust toward clients, stereotypes and assumptions about customers, and financial incentives.

**Conclusions/Significance:**

These findings suggest that social and economic factors play an important role in shaping sexual decision-making among female sex workers in Guangzhou. We argue that greater insight into and attention to these factors could enhance the success of HIV prevention efforts.

## Introduction

Since the socio-economic reform and opening-up policies were introduced in 1978, China has experienced increased economic growth. After almost 30 years of constrained mobility, an estimated 120 million migrants have relocated to rapidly changing cities [1]. According to the fifth Chinese census, there were 12.7 million residents in Guangzhou in 2010, one-third of whom were migrants. Among this migrant population, 45.1% were female [2]. A lack of employment opportunities that provide adequate salaries to migrant women in cities may compel some to enter the commercial sex industry. A growing number of women who cannot find a job or earn enough to support their families are thus attracted by the profit potential of the sex industry. Research among FSWs also indicates that most are migrants [3]–[5].

Heterosexual transmission of HIV has increased in recent years and has become the major driver of the HIV epidemic in China. According to the estimates found jointly by China’s Ministry of Health, UNAIDS, and World Health Organization in 2011, there were approximately 780,000 people living with HIV/AIDS at the end of 2011; heterosexual transmission accounted for 46.5% of these cases [6]. The results from HIV sentinel surveillance show that HIV prevalence among sex workers has increased in recent years [7]. In Guangdong, where this research was based, HIV prevalence among FSWs was estimated to be approximately 3% and rising [8]. Accompanying the rapid increase in the HIV epidemic, the STIs (sexually transmitted infections) epidemic is severer among FSWs. A community-based study of 966 commercial sex workers in Guangdong province found that 14% were infected with syphilis, 32% with Chlamydia, and 8% with gonorrhea [5]. Another study showed that the growing commercial sex industry can be closely linked to the spread of STIs [9].

Sex workers in China continue to engage in unprotected sex acts that put them at risk for contracting HIV and other STIs. Small behavioral surveillance studies in China have also found that consistent condom use was low with paying customers [4], [6], [8], [10], [11] and was lower with non-paying or more regular partners [8]–[10]. One large study of FSWs in Shenzhen reported consistent condom use to be 80% with clients but only 10% with non-commercial partners. [10] Other studies have reported similar levels of condom use in commercial relationships [8], [12], [13] and lower levels of consistent condom use in non-commercial relationships (17–24%). [8], [12].

Many published quantitative and qualitative studies have investigated barriers to condom use among diverse FSW populations. Inconsistent condom use among China’s FSWs has been associated with lower age, lower educational attainment and higher number of clients per day [8], [10]. Several studies have identified social influences, such as trust in a stable partner [4], sex venue [14], [15], occupational characteristics [13] and social support [14], [16] as important determinants of unsafe sex among FSWs.

The Theory of Planned Behaviors (TPBs) has been used to investigate a wide range of health-related behaviors [17]. The model specifies that behavioral intention (perceived likelihood of performing the behavior) is an important determinant of health-related behaviors, which is in turn determined by relevant attitudes (overall evaluation of the behavior), social norms (belief about whether most people approve or disapprove of the behaviors), as well as perceived behavioral control (an individual’s assessment of the ease or difficulty of performing a given behavior). The theory of planned behaviors was proposed as a theoretical framework [18] to explore in greater detail STI- and HIV-related risk behavior of FSWs and barriers to condom use among FSWs recruited from the community in the city of Guangzhou, China. Qualitative research is starting to show that strong social and cultural forces shape sexual behavior. This research also helps to explain why providing information and condoms–while important–is often not enough to change behavior. Specifically, such work helps us understand why some HIV prevention programs have been ineffective and how they might be improved.

## Methodology

### Research Sites

Guangzhou, which neighbors Hong Kong in southern China, is one of the most rapidly growing cities in the world. The city hosts a thriving commercial sex industry attracting local consumers and consumers from Hong Kong and abroad.

### Design

Given the stigma attached to sex work, it is difficult to effectively measure sensitive subjects, such as HIV risk and condom use, using survey methods. In an attempt to limit desirability bias and to investigate the explanations behind low condom use, we undertook an exploratory qualitative study involving qualitative methods and interviewed 24 women working in the sex industry.

### Instrument Development

This choice of framework guided our decision to include key areas for exploration in line with the study aims. Study domains were discussed and agreed upon by all the authors in this study; then, JW drafted the topic guide. The topic guide included the following: (1) basic socio-demographic information; (2) living status and reasons for entering the sex industry; (3) health-seeking behavior; (4) knowledge of FSWs; (5) sexual behavior; and (6) barriers to condom use. Our research team included native Mandarin and Cantonese speakers. We tested the guide during five interviews with FSWs.

### Sampling and Recruitment

To achieve the aims of the qualitative study, we purposively selected from a wide range of sample groups to facilitate the emergence of a diversity of views and perspectives. Our outreach staff set out to interview a maximum variation sample of community-based FSWs. Existing contacts among members of the research team with FSW peer educators, relations between FSWs themselves, and other people having regular contact with FSWs–including a health educator and HIV counselor–were used to approach FSWs. In addition, data collectors approached FSWs on streets or venues where they frequently congregated. Women were purposefully selected based on pre-specified categories defined by their type of work and employment status (more details presented in Table 1). Inclusion criteria were: being over 18 years of age and admission of sex work. Twenty-eight FSWs were approached by the local CDC (Center for Disease Control and Prevention), and 10 FSWs were introduced by the health educator and HIV counselor.

**Table 1:** Categories of Female Sex Work.

Categories	Name	Organization of Work and Employment Status	Income	Demographic Characteristics
1	Second wife (e’ nai)	Hired for a period by one client Self-employed	High & stable	Variable education
2	Courtesan	Big hotel, VIP club Self-employed	High	Well educated, young
3	Karaoke dancing girl	Big karaoke and dancing bar Employed by manager	Middle	Middle-level education, young
4	Massage girl (anmo nu)	Massage parlor Employed by manager	Middle	Middle-level education, young
5	Beauty Parlor girl (falang xiaojie)	Work in barbershop, small singing dancing bar, or smallroadside restaurant. Employed by manager or pimp	Middle-low	Middle-low education, young-middle age
6	Streetstanding girl (zhanjie nu)	Solicit in street, park. Employed by pimp or self-employed	Low	Low education, tend to be older
7	Factory girl (gongpeng nu)	Solicit in construction field or small factory. Employedby pimp	Lowest	Low education, tend to be older

Adapted from [36].

### Study Procedure

We explained the aims and nature of the study, assuring women that participation was voluntary. Participants were guaranteed confidentiality and anonymity and were informed of their right to end the interview at any time if they felt any discomfort. Participation was voluntary with no direct benefits, except honorariums of RMB100 (approximately 15 US dollars). By comparison, workers usually charge RMB200 (about 30 US dollars) for commercial sexual transactions. Written informed consent was obtained from every respondent before the interviews. Respondents participated in face-to-face, semi-structured, in-depth interviews that lasted approximately 40–50 minutes. The author conducted the interviews in the women’s place of work or their homes (private room).

### Ethics

The research protocols were reviewed and approved by the Institutional Review Board at the School of Public Health of Sun Yat-Sen University. No identifying information was collected as part of this research. The data were stored at the School of Public Health of Sun Yat-Sen University in accordance with the China Data Protection Act.

### Data Management and Analysis

All interviews were recorded and later transcribed into written Chinese. After reading the interview transcripts, field notes and participant observation reports in full, a coding framework was developed, which included social demographics, living status and reasons for entering the sex industry, health-seeking behaviors, knowledge of HIV and STIs, sexual behavior, and barriers to condom use. The transcripts and formal summary reports of field notes were then imported into NVivo 8.0 and coded line-by-line. Two coders were involved in excerpting, coding and analyzing the qualitative data. On completion of coding, the first author conducted comparative analysis across the themes to explore issues related to condom use and other relevant themes.

## Results

### Socio-demographic Characteristics

All 24 subjects were of Han Chinese ethnicity and aged 18 to 32 years (mean age 23.5 years). One-third of the subjects (8/24) were married. The majority (20/24) said they had received less than 12 years of education (senior high school education). Four had senior high school education, 12 junior high school education, and 8 elementary education. All but 4 were born in mainland China in small towns or rural villages. The most common reason given for migrating to Guangzhou was to pursue economic opportunities. The charge for a normal commercial sex transaction ranged from 20 ($3) to 1000 RMB ($150). The median was 200 RMB (about $30). Detailed demographic information for the participants can be found in Table 2.

**Table 2:** Demographic Characteristics of FSWs’ participants.

ID	Age (years)	Marital status	Level of education	Venue type#	Place of interview
1	24	Married	elementary educations.	4	work place
2	19	Unmarried	junior high school	5	work place
3	26	Married	senior high school	5	work place
4	23	Unmarried	senior high school	5	work place
5	21	Unmarried	junior high school	5	work place
6	18	Unmarried	junior high school	5	work place
7	24	Unmarried	elementary educations.	6	their home
8	23	Unmarried	elementary educations.	4	work place
9	27	Married	junior high school	6	their home
10	25	Married	elementary educations.	5	work place
11	20	Unmarried	junior high school	3	work place
12	19	Unmarried	elementary educations.	4	work place
13	21	Unmarried	senior high school	5	work place
14	32	Married	junior high school	6	their home
15	22	Unmarried	junior high school	4	work place
16	22	Unmarried	senior high school	3	work place
17	19	Unmarried	elementary educations.	4	work place
18	18	Unmarried	junior high school	4	work place
19	26	Married	junior high school	5	work place
20	24	Unmarried	junior high school	5	work place
21	25	Unmarried	junior high school	4	work place
22	25	Unmarried	elementary educations.	4	work place
23	30	Married	elementary educations.	6	their home
24	31	Married	junior high school	5	work place

# Classification based on table 1.

### Living Status and Reasons for Entering the Sex Industry

Women were asked about their current living status and if they wanted to continue working in the sex industry. The majority indicated that they were not satisfied with their current living status. Almost all expressed concerns with health problems caused by drinking alcohol with clients. All but one expressed a desire to leave the industry, which was unsurprising because many have been recently incarcerated and considering the cultural context. However, none of the women had concrete plans for abandoning sex work in the near future. They said they would leave the industry when they earned ‘enough money’; others were committed to leaving‘next year’, ‘at the start of the Spring Festival’, or ‘when I find a good job’. Many reported that they were trying to earn enough money to return to their hometowns and explained that they would ‘lose face’ if they returned home from their migration penniless.

When I first entered the sex industry, my goal was to earn a lot of money and go back to my hometown. But if I do not return with lots of money, why should I enter the damned sex industry? [ID 11, 20 years old, unmarried]

From my own prospective, a lot of money means I can start my own business, such as running a small shop or restaurant, and not fear being fired or losing my job. [ID 9, 27 years old, married]

All except one respondent reported entering the sex industry for financial reasons following unsuccessful attempts at establishing themselves in other industries. Often, those interviewed did not refer to sex work. Instead, they used the commonly accepted euphemism, “work in recreational venues.”

I came to Guangzhou in August 2006; before that I worked in Henan province. My previous work was not in recreational venues, I used to work in a restaurant as a waitress. My co-worker told me, it’s very easy and quick to earn a lot of money in Guangzhou. At that time, my monthly income was about 800 RMB (about $120), the majority of which I saved to send to my parents who are farmers. There was little left over for myself. [ID 13, 21 years old, unmarried]

Before I came to Guangzhou, I sold mobile cells for a boss. The monthly income was very unstable; sometimes I earned only 500 RMB (about $80). You know, girls always like shopping; however, this little money could not cover my basic livelihood, let alone shopping. One of my best friends told me that being a sex worker in Guangzhou was an easy way to earn lots of money. So I came to this venue. [ID 16, 22 years old, unmarried]

Some women knew exactly what they would be expected to do while working in recreational venues; however, half the women told stories of being lured into sex work by their ‘boyfriends’ or sex-worker friends and claim that they were unaware of the work they would be expected to do:

When I first came to Guangzhou, I worked in a small restaurant. One day, a friend invited me to join a party being given by an elder sister who was also from my hometown. At that party, she told me she also had a restaurant and that I could earn more than I was at the time, so I came to work for her without thinking. However, when I arrived at what she called a restaurant I learned she was actually a *mami* (an intermediary between sex workers and customers). [ID 21, 25 years old, unmarried]

Another interviewee explained that more generally, women find themselves with little choice but to enter the sex industry due to financial failure.

When I first came to Guangzhou, I worked in a factory that produced shoes. At that time, my monthly income was approximately 1000 RMB ($150). Frankly, my income was enough to cover my daily expenses, however, in my hometown I have two brothers who are still in school. My father died when I was 10 years old. My mother farms to support the whole family. The salary from the factory was not enough to help my mother to support my little brothers. Even so, when my friend advised me to go into sex work, I took a long time to follow her advice. Actually, as far as I know, there are several women from the factory where I used to work who have since entered the sex industry as well. [ID 7, 24 years old, unmarried]

### Health-seeking Behaviors

More than half (17/24) had not been tested for HIV because of the following reasons: (1) they believed they had taken sufficient measures to keep themselves “safe”; (2) because prostitution is illegal in China, they feared that taking an HIV test would make it easier for police to track and arrest them (actually, police cannot use being tested for HIV as evidence to arrest them); (3) because of the potential stigma related to prostitution, they did not want to disclose their occupation to others, including medical professionals.

An ordinary girl will never go to an STI clinic, only those girls who have multiple sex partners will get an STI and have to get medicine in an STI clinic. I used to go to an STI clinic, but when I saw the majority of patients waiting for treatment were male, I decided to leave because I didn’t want to shame myself. [ID 10, 25 years old, married]

I feel very shameful to go to a hospital to get STI medical services. You know, for a girl, presenting yourself in an STI-specific clinic means you are a dirty girl. When I have symptoms of an STI, I prefer going to a pharmacy to get medicine to treat it. [ID 13, 21 years old, unmarried]

### Sexual Behaviors

Of those we interviewed, approximately two-thirds had‘boyfriends’ or a husband at the time of the interview, and the majority (22/24) had been sexually active before entering the sex industry. Most (17/24) reported they had used a condom at first sexual intercourse.

Only two women reported always using condoms with their husbands or boyfriends. Those who did not use condoms with boyfriends or husbands explained this was because they wanted to maintain closeness and domestic stability, or because their boyfriends/husbands did not like condoms. Such views are also articulated as follows:

When I have sex with my boyfriend, using a condom can reduce our intimacy. He is my beloved; how could I have sex with him with a condom? Condom use can also reduce the trust between us. [ID 02, 19 years old, unmarried]

My boyfriend doesn’t like to use a condom when we have sex because he doesn’t know I am a sex worker. If I asked my boyfriend to start using condoms he would suspect me of having had unprotected sex with other guys. [ID 11, 20 years old unmarried]

All of the women reporting using condoms when having sex with clients, but only five reported always doing so.

Normally, I will use a condom when I have sex with a client, but when clients pay more money for having sex without condoms, I also feel ok to do that. [ID 13, 21 years old, unmarried]

Under some circumstances, if some clients insist on not using a condom, I have no choice but to compromise. After all, I came here for the money. [ID 19, 26 years old, married]

### Knowledge of HIV and STIs

Those interviewed seemed to have very limited knowledge about HIV and STIs. Although all knew that HIV could be transmitted by sex, less than half could accurately name the major routes of HIV transmission and effective preventive measures.

I know a little about AIDS, it is a kind of incurable disease. I mainly use potassium permanganate (An inorganic chemical compound with the formula KMnO4, which is usually used as an antiseptic. It has no known protective effect against STIs.) to prevent myself from getting such diseases (STIs). Every night when I come back to my home, I use potassium permanganate to wash down my body. I was advised to do this by one co-worker. [ID 10, 25 years old, married]

All but three respondents believed that sex workers were at high risk of acquiring HIV. One woman felt FSWs were at lower risk than the general population and two were unsure. However, only two women felt personally at risk. One reported always using condoms with clients and with her boyfriend; the other reported only sometimes using condoms with clients. The other 19 respondents framed HIV as something ‘caught’ by others and explained that HIV was ‘far from them’. Some explained their low-risk perceptions through reporting regular condom use behavior and taking other steps to protect themselves (e.g., potassium permanganate).

### Barriers to Condom Use

Factors discouraging condom use included wanting to demonstrate their trust toward boyfriends and husbands, but also toward customers; personal assumptions and feelings toward regular partners; and financial incentives.

#### Unreasonable trust toward clients

In general, women seemed willing to believe men, whether customers or regular partners, who claimed they were “safe.” While this trust might be interpreted as blind, there are several factors at stake that complicate the situation. Few of the women could manage to negotiate consistent condom use with customers, despite their reported desire to do so. This seems to stem largely from the fact that many men were willing to pay more money for sex without a condom. However, as one respondent noted, the nature of their work requires them to please the customer, and many customers simply do not want to use condoms. The men are not inclined to worry about their own health despite the fact that they are paying for sex, which perhaps presents a more perplexing question than why the women are not using condoms. Another factor is that there is always the possibility that a client may become a boyfriend and perhaps even a husband. Given this possibility, women want to show they trust the men, but also that they are trustworthy as potential mates. Moreover, it seemed that the norm of condom use was abandoned when clients became regulars and came to be seen as friends who were perceived as “safe”. Half (12/24) of the women said they had stopped using condoms with regular clients, often when they felt the relationship was well enough established. There was no set timing for this, with condoms being stopped after anything from a few weeks to almost a year. Condom discontinuation was regarded as a demonstration of trust:

I usually use condoms with new clients. I seldom use condoms with those who I have had sex for many times, since I am familiar with those guys. I know their health status and they know me as well. We’re quite safe with each other. In addition, those familiar clients often have a relationship with our *mami*. If I insist on using condoms, they (clients) will complain to her. [ID 17, 19 years old unmarried]

#### Stereotypes and personal assumptions

Similar to decisions made to abandon condom use with regular clients, a couple (2/24) reasoned that they could assess potential sexual partners as “clean” or “unclean”. This reasoning seemed to be entirely based on stereotypes and personal assumptions about both HIV and the appearance of clients, which linked dirt, poverty, and rural background to HIV risk.

When I have sex with farmers (farmers who have migrated to the city for work), I absolutely use condoms. They look very dirty and I am not sure whether they have a disease. However, if my clients are white-collar, sometimes I may have sex with them without condoms. Because these guys are very concerned about their health, they seldom get diseases. Besides, they are cleaner, even having sex with them without a condom does not make me feel uncomfortable. [ID 03, 26 years old, married]]

#### Financial reasons

Another major barrier to condom use involved financial incentives. FSWs reported that some clients offer more money to have sex without a condom. One woman reported that she earns double when she foregoes a condom.

Some clients don’t like to use a condom so they usually pay twice to ask me not to use a condom. You know, the reason why I entered this industry was just for money, so I have no reason to refuse to do that. [ID 13, 21 years old, unmarried]

As all of the women reported becoming sex workers for financial reasons, it makes sense that money would also be an incentive to forego condom use. As migrants, they can usually find jobs that will allow them to cover the costs of their basic survival in the city, but the reason they came to the city was to help relieve economic hardship felt by their families in the rural areas. If they want to avoid being seen as failures, they are expected to send money home to support parents and grandparents and to pay siblings’ tuition fees, as well as family debts incurred from misfortune, including natural disaster and illness. We found that it was often when women were faced with dire financial situations that they turned to sex work, and that being economically marginalized, they were rarely in a position to refuse more money for having sex without a condom.

## Discussion

Little research has been conducted on factors that affect FSWs’ condom use using qualitative methods in China. This research illustrates that a desire to promote trust, combined with stereotypes and personal assumptions about partners, and financial incentives were the main reasons women gave for agreeing to have sex without condoms.

### Health-seeking Behaviors

The low reported HIV testing rate reflects constraints in accessing medical services due to the potential stigma attached to FSWs. The women’s decisions to seek HIV testing services are influenced by the complex interactions of personal risk assessment, interpersonal and social relationships, and social stigma toward FSWs [19]. Our study also indicates that personal risk perceptions (e.g., they believed they had taken sufficient measures to keep themselves “safe”), combined with pervasive social stigma toward FSWs, hinder their access to and use of available services for HIV and STI testing and treatment. To address this issue, social scientists, health educators and NGOs (non-governmental organizations) should work together to launch campaigns to destigmatize STI health-seeking behaviors as well as to enhance personal risk awareness among FSWs.

### Knowledge of HIV and STIs

Knowledge of HIV and STIs exerts a major influence on FSWs’ perceived risk, attitudes and behaviors, affecting their condom use and other preventive practices [19]. In our study, FSWs demonstrated limited knowledge of HIV and preventive measures against HIV and STIs. For instance, some subjects use potassium permanganate to prevent HIV and STIs. This finding suggests that HIV/STI prevention information dissemination among FSWs in China should be strengthened.

### Barriers to Condom Use

Adequate access and consistent use of condoms are fundamental to the prevention of HIV and STIs. Women were aware that proper condom use is an effective preventive measure for HIV. Unfortunately, condoms were not always used either with their intimate partner or clients. This was not because of high prices or difficulties in procuring condoms. In our study, a desire to promote trust, combined with stereotypes and personal assumptions about partners, and financial incentives were the main reasons women gave for agreeing to have sex without condoms.

Knowing and trusting a client has been cited as a key barrier to condom use in China [20], [21] and elsewhere [22]–[24]. Chinese culture emphasizes interpersonal trust as a product of familiarity, reciprocity and the degree of intimacy [25]. Research among heterosexuals has demonstrated that, particularly among young women, trust is central in defining the meaning of sexual involvements. In relation to safe sex, young women, and sometimes men, often “trust to love,” in that they see condom use as unnecessary with a regular partner or within a relationship [11], [26]. Furthermore, these women articulate love and trust as a prophylactic, and sex is constructed as safe through its relationship with love [27]. Our data support the view that decisions about condom use are partner-specific [28] and that the nature of a relationship, i.e., degree of acquaintance as well as means of acquaintance, plays a key role in determining whether condoms are used [9]. To address this issue, systematic training should be provided to educate FSWs that trust cannot protect them from contracting HIV and STIs.

Studies repeatedly showed that young people assess the disease risk of a potential partner by how well they know their partner socially, their partner’s appearance, or other unreliable indicators [29]–[33]. They readily use condoms to protect against disease with “risky” partners, however, when it comes to partners they deem “safe”, they often compromise. A study in Nepal reveals FSWs were relaxed and tended not to use condoms with apparently healthy, young, educated and wealthier clients [34]. Our study also found that FSWs tended to make condom use decisions based on their own stereotypes and personal assumptions about partners. The finding can be used to help answer specific questions, for example, why some young people are inconsistent condom users, even with high levels of knowledge and access to condoms. Young people may choose not to use a condom with a partner they perceive to be “clean”. This indicates that greater effort should be made in prevention campaigns to de-link HIV with “dirtiness”. The message needs to be that anyone can get HIV, farmer or business man, prostitute or professor.

FSWs who entered the industry voluntarily did so with the purpose of making money [13]. Making money offers a lifestyle of comfort, but perhaps more importantly, it confers a sense of ‘face’ or respect to the person who has become financially independent – and thus able to help one’s family financially. Losing out financially means far more than economic hardship; it is a threat to self-identity [32]. However, the desire to make more money can lead to a significant difference in the decision regarding condom use. Economic barriers and lack of negotiation skills are considered two major factors that discourage condom use with clients, even though FSWs have a high level of HIV knowledge [35]. In this context, the negotiation skills for condom use are crucial, as clients tend to be willing to pay more for intercourse without protection. Condom promotion strategies should be directed at the community to address sex workers’ economic barriers to condom use, and at the personal level to develop sex workers’ negotiation skills. The latter would not only empower sex workers to successfully get clients to use condoms when brothel support is lacking, but it would also increase their sense of control over their work situation and hence prepare them for future empowerment and reorganization efforts to improve their self reliance and welfare. [35].

There are limitations to this study. FSWs are difficult to locate due to stigma as well as illicit status, so non-probability convenient sampling was used for recruitment. The small sample size, with findings based on self-reported data, potentially implies memory bias and information concealment. The financial incentive, although small, might have led some respondents to expect more resources in the future, possibly affecting their responses. Our study was not designed to test predictors of condom use; rather, our results will be used to design and evaluate an appropriate intervention to increase condom use by FSWs in Guangzhou.
